# Mediating factors of coping process in parents of children with type 1 diabetes

**DOI:** 10.1186/2251-6581-12-20

**Published:** 2013-05-14

**Authors:** Fatemeh Oskouie, Neda Mehrdad, Hossein Ebrahimi

**Affiliations:** 1 Center for Nursing Care Research/School of Nursing & Midwifery, Iran University of Medical Sciences, Tehran, Iran; 2 Endocrinology and Metabolism Research Center, Endocrinology and Metabolism Research Institute, Center for Nursing Care Research/School of Nursing & Midwifery, Tehran University of Medical Sciences, Tehran, Iran; 3 School of Nursing and Midwifery, Shahroud University of Medical Sciences, Shahroud, Iran

**Keywords:** Children, Coping, Grounded theory, Mediating factors, Parents, Type 1 diabetes

## Abstract

**Background:**

Type 1 diabetes is a lifelong condition for children and their parents, the management for which imposes a vast responsibility. This study explores the mediating factors that affect Iranian parents’ coping processes with their children’s type 1 diabetes.

**Methods:**

Research was conducted using the grounded theory method. Participants were selected purposefully, and we continued with theoretical sampling. Constant comparative analysis was used to analyze the data.

**Results:**

The mediating factors of the parental coping process with their child’s diabetes consist of the child’s cooperation, crises and experiences, economic challenges, and parental participation in care.

**Conclusion:**

Findings highlight the necessity of well-informed nurses with insightful understanding of the mediating factors in parental coping with juvenile diabetes in order to meet the particular needs of this group.

## Introduction

Type 1 diabetes is a chronic disease whose prevalence is increasing throughout the world [1]. It is also a lifelong situation that invades the lives of children and their parents [2], the management of which imposes a huge responsibility on children and their parents [3], especially in young children [4].

Although parents of these children may experience stressful and difficult conditions [5], their participation in diabetes management has consistently been considered as an important indicator of positive health outcomes among children. Moreover, high-quality metabolic control in children with diabetes is related to the coping of parents and the management of their emotional responses [6]. The manner of parents in the daily management of the disease is an important factor in promoting their children’s glycemic control [7,8]. In order to cope with and manage problems brought about by the disease, parents employ different intervening events or conditions as mediating factors [9] (defined as factors that moderate or otherwise alter the impact of causal conditions on phenomena [Strauss and Corbin 1998]). Several studies have focused on parents’ coping with their child’s diabetes, including phases of coping, coping strategies, and parents’ psychological and emotional responses to their children’s diabetes [10-12]. In an early study by Seppanen and colleagues (1999), six phases of parental coping with their children’s diabetes were outlined: disbelief, lack of information, and guilt, learning to care, normalization, uncertainty, and reorganization. That study reported different parental experiences of stress, coping strategies, and sense of control in each of these phases. The coping strategies that were identified in the study by Damião and colleagues (2009) consist of distancing, escape-avoidance, social support, accepting responsibility, problem solving, and positive reappraisal. Parents had adapted to the needs of diabetes management, but most of them experienced reappearances of grief at critical times even though 7–10 years had passed since their children’s diagnoses [10].

Psychological, emotional, and social factors play important roles in chronic illness outcomes. The potential barriers to healthy coping are numerous. Some of them consist of low social support, financial stress or constraint, external locus of control, lack of access to providers and diabetes educators, low problem-solving ability, and stressful life events [13]. Although parents have a basic role as caregivers, little is known about the mediating factors that affect parental coping with their children’s diabetes.

Studies in Iran have focused on issues such as parents’ worries [14] and their emotional reactions to the diagnosis of diabetes in their children [15]. Therefore, because of limited information about parental coping with children’s diabetes and a lack of knowledge about related mediating factors in the cultural setting of Iran, this study was designed.

The aim of this study was to identify mediating factors affecting Iranian parents’ coping processes with their children’s type 1 diabetes.

## Methods

This qualitative study was completed as part of a doctoral dissertation and is based on grounded theory. Grounded theory “focuses on the identification, description, and explanation of interactional processes among individuals or groups” [16] and makes it possible to deeply study clinical actions, behaviors, beliefs, and attitudes just as they occur in real life [17].

Seventeen informants were participants in this study. We initially used a purposive sampling procedure, but while the research continued, we began to use theoretical sampling on the basis of concepts that emerged from the data. Theoretical sampling proceeded until the attributes and dimensions of the categories were complete [16].

The participants comprised 15 parents (eight fathers and seven mothers) of children with diabetes who lived with their children; their ages ranged 28–51 years. Their children had been diagnosed with diabetes 5 months–11 years previously and were aged 8–18 years. Two other participants were women: a 22-year-old woman with an 8-year history of diabetes and a 32-year-old woman who was scientific director of a diabetes association with five years of experience.

The study was approved by the Ethics Committee of Tehran University of Medical Sciences (Project Number: 90/250.5825, Approval Date: 2011/12/12) and has been done in the Diabetes Center of Shahroud University of Medical Sciences and the Gabric Diabetes Association in Tehran. The participants were informed of the purpose of the study, the institute undertaking the research, the confidentiality of the study participants, and the fact that they could withdraw from the study at any time.

Data were collected using semi-structured interviews in the participants’ choice of setting. The interview guide consisted of open-ended questions to allow the respondents to clarify their own experiences. After a warm-up dialogue with each participant, data collection was initiated, and overarching, prompting questions were used whenever needed. The mean duration of the interviews was 66 minutes. Field notes were also taken, and immediately after each interview, the main points that arose during the interview and were included in field notes were written down, transcribed, and analyzed. On the basis of each analysis, the next participant was chosen.

We stopped carrying out interviews when saturation of the categories occurred. Data were collected and analyzed from January 2010 to August 2011.

The interview started with the question, “How were you informed of your child’s diabetes?” More explicit questions followed the early questions, allowing the researcher to discover the issues that had been raised by the participants in previous interviews. For example, when participants said that their child’s diabetes changed their normal life patterns, we instructed them, “Tell me about your daily living after you found out about your child’s disease.”

Constant comparative analysis and three stages of open coding, axial coding, and selective coding were utilized [18,19]. Each interview was analyzed and coded before starting the next interview. In order to do open coding, the transcripts were checked out word by word and line by line, and codes were allocated to repeated themes, reflecting the respondents’ own words. Codes with similar meanings were arranged and categorized by making comparisons to define the features of each concept. Categories were connected to their subcategories and linked at the level of their characteristics and dimensions, a process called axial coding. At this stage, merging and purification of the theory was obtained by selective coding.

We assured credibility by using various sources of the same information, including numerous interviews, and these were used to validate the findings (data triangulation). The researcher devoted enough time to collect the data through constant presence in the field and immersion in the data (prolonged engagement) [19]. Two auditors checked the study process and examined the analysis process and records for accuracy. The codes and categories were checked by the two other researchers on a regular basis, and consensus decisions were made (dependability) [20].

After explanation of the phenomenon was complete, it was rechecked by nine participants (confirmability). Transferability can be judged by readers examining the original context, participant individuality, data collection and analysis, and the quotations included in the method and results sections [20].

## Results

**Figure 1:**
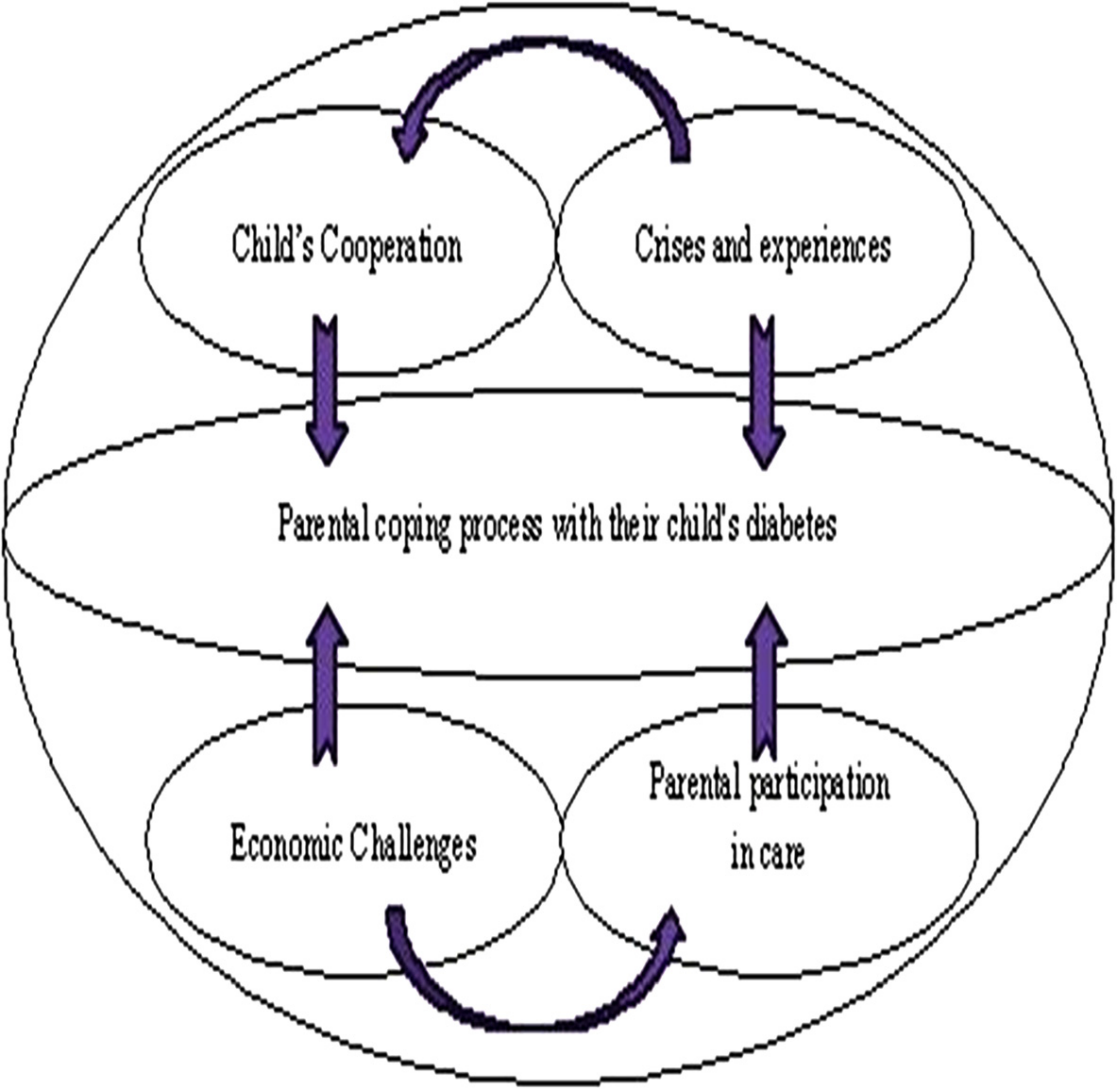
Diagrammatical representation of the mediating factors in parental coping process with their child's diabetes.

In this study, mediating factors in parental coping processes with their children’s diabetes consist of four main categories: “child’s cooperation,” “crises and experiences,” “economic challenges,” and “parental participation in care” (Figure 1).

### Child’s cooperation

Cooperation of the child with diabetes treatment has a positive impact on the parents’ coping process, which depends on the patients’ characteristics (including age and gender), understanding, and empowerment.

#### Patients’ characteristics

The third participant said, “*my son cooperates, but in a lesser way, because he is still a very young child.*” In addition, parents were concerned about their children’s lack of cooperation when they reached adolescence and thereafter. “*Currently, his behavior and actions are appropriate, but when he grows up, during the adolescent period, he might not cooperate with taking insulin*” *(P4)*. “*A little girl differs from a boy having a certain physical condition and expressing jealousy, aside from being delicate*” *(P7)*.

*Understanding and empowerment:* patients’ understanding and capabilities in managing their diabetes are the factors that had positive impacts on parents’ coping with their children’s diabetes. Parents also pointed out that lack of cooperation on the part of the patients, specifically in the early months after diagnosis with the disease, resulted in exacerbations of their problems. “*If my child’s perception of the situation were very poor, then it would put so much pressure on me*” *(P12).* “*In the first months after diagnosis, my child never allowed me to administer her insulin injection*” *(P2).* “*At the time of diagnosis, the main concern of my parents was that I would not do anything to control my blood sugar*” *(P13).*

### Crises and experiences

Crisis and experiences were the results of the two subcategories of “situational crises” and “hard experiences.”

#### Situational crises

The occurrence of a situational crisis in a family, such as puberty in a diabetic child or terminal illness of a family member, usually has a negative effect on the child’s metabolic control and recreates problems involved with parental coping with their child’s illness. The first participant said: “*Her glucose level has gone up; they said that this is because she is reaching puberty.*” Another participant said: “*During her father’s illness, there was too much pressure on her and me*” *(P2).*

#### Hard experiences

Despite the fact that some parents have overcome the initial shock and the hardships brought about by their children’s diabetes and have come to terms with the illness, this ability to cope was not strong enough to cope with the additional tension that resulted from difficult events. “*After her (my diabetic child) liver disease, she became very irritable and bad-tempered*” *(P15).* Past tricky experiences affected how they would cope with new crises. One participant reported the following regarding her daughter, who was already suffering from thyroid cancer: “*Really, diabetes is much worse than cancer: my daughter is able to fight with cancer, but diabetes is different; you could never bear it*” *(P9).* Another participant said, “*When I understood that my child has diabetes, I remembered that I have two brothers who were martyred; I think these experiences have helped me very much to cope with my child’s illness*” *(P10).*

### Economic challenges

Economic challenges were the result of the two subcategories of economic condition and costs of treatment.

#### Economic condition

expensive treatment for diabetes was a barrier in providing the necessary requirements for children to maintain optimum metabolic control in families that were less financially endowed; in turn, this results in stress and anxiety among the parents. “*Diabetes is really a disease for the rich… when parents do not have the money to buy a pack of glucose test strips, how can they control their child’s blood sugar?*” *(P13).* Another participant said, *"Since it is expensive, I cannot afford to check the blood glucose of my daughter often enough*” *(P9).*

#### Costs of treatment

The cost imposed by a diabetic child, its effects on other aspects of life, and the shortage of support from insurance companies were other factors mentioned by the participants. “*The cost was an expensive addition to our household budget… Therefore, we tried to cut down on other expenses in order to make ends meet*” *(P5).* “*The insurance system pays for the laboratory examinations, but other expenses are paid by me*” *(P4).* In order to reduce expenses, parents have minimized their control of their children’s blood glucose levels; therefore, this issue influences their coping with their child’s diabetes. “*Initially, we checked her blood glucose at least twice or three times per day, but at present, we only check her glucose once every three days, or even once a week*” *(P7).*

### Parental participation in care

Parental care seemed to fit into the two subcategories of “dominant maternal roles in the home” and “role of fathers outside the home.”

#### Dominant maternal roles in the home

More responsibilities were declared by all participants to be shouldered by mothers in caring for their diabetic children, and their motherly nature has been indicated as the reason for mothers’ significant roles in taking care of their diabetic children. A father said, “*My wife is more involved in taking care of our child, and my absence is the reason why my wife’s responsibility in taking care of our diabetic child has been doubled*” *(P5).* A mother said, “*I believe that a father cannot cope with cooperating, especially on this issue*” *(P12).*

Although the mother’s role has been seen as more significant, mothers who were satisfied with the husband’s participation in rendering care to their children believed that this participation is an important factor in restoring peace and calm. “*I think we shouldered the same amount of responsibilities, but since I am a housewife, maybe I could spend more time with our diabetic child*” *(P16).* Of course, being alone in taking care of a sick child is very difficult and stressful, as stated by majority of the mothers. “*I think that in all my life, I have never experienced such difficulty as this; it was a very terrible time, and it caused the feeling that I was alone*” *(P12).*

#### Role of fathers outside the home

The reasons for fathers’ less-significant roles in taking care of diabetic children at home consist of their lack of presence at home because of their work, their lack of knowledge regarding diabetes, and their fear of the occurrence of potential problems during their interventions. Fathers said, *“Since most of my time is spent in my workplace, I have no idea what to do to care for my diabetic child*” *(P14).* “*I don’t interfere with anything because my wife has given her full attention to caring for our child, and there is no need for me to intervene*” *(P15).* Moreover, the majority of the fathers believed that the mother’s presence is enough to solve the problem and evade any duties at home. “*My husband himself tries to avoid these types of problems, meaning that he does not want to get involved in these kinds of issues*” *(P2).*

Taking into consideration the Iranian cultural background, in which the father is considered to be the family’s breadwinner and has to work outside to earn a living, and in which most household responsibilities are entrusted to mothers, it seems that most parents believed that caring for a diabetic child is considered to be a household matter. Therefore, the mother has greater responsibilities and needs to use more strategies to approach the problems.

## Discussion

The findings of this study are that parents coping with their children’s diabetes are affected by the patient’s cooperation, crises and experiences, economic challenges, and parental participation in care.

We found that children’s cooperation was influenced by their characteristics, their understanding, and their empowerment. Concept analysis revealed that the child’s age, nature, and the severity of his/her illness affect the family’s ability to return to normal status [21]. The effect of the diagnosis of juvenile diabetes on the family varies depending on the child’s age and the family’s socioeconomic status [22]. Research findings have suggested that parents worry about the lack cooperation on the part of their children when they reach adolescence and thereafter. Several studies have indicated that psychological factors (such as stressors and coping patterns) that arise during adolescence are often associated with negligence in their self-management [23]. Moreover, findings have revealed that a diabetic child’s gender affects child–parent cooperation and Parental coping. Grootenhuis (1997) stated that mothers with diabetic sons are most affected, and higher rates of depression among them have been reported. A study from Iran showed that inappropriate community and family responses were some of the reasons for parental concern about their daughters’ chronic illness [14]. Therefore, gender differences between male and female adolescents with diabetes require special attention from family and care providers.

Our research findings indicated that an increase in the patient understands of, empowerment regarding and participation in the management of diabetes is related to better parental coping with their child’s diabetes. Whittemore and colleagues (2003) believed that most families with school-aged children are able to manage diabetes well enough, but the burden placed upon parents can exacerbate depression symptoms, and caring for the child may be accompanied by important consequences [24]. Studies about parent-adolescent interaction to manage disease indicated that this interaction is related with adherence to treatment [25]. Since the parental coping process is influenced by various factors, it seems to be reinforced by positive features such as support and warmth, and cooperation between family members can facilitate the coping process with their child’s diabetes.

The results of our study revealed that crises and experiences are other mediating factors that can affect parents’ coping with their children’s diabetes. For example, high blood sugar levels due to the child’s puberty were one reason for parents’ worries. Ivey and colleagues (2009) stated that as the adolescent tries to create a sense of individual identity and independence, it is usual for conflicts to happen, and these conflicts repeatedly center on routinely recurring events. Debate related to illness management can bring about supplemental and exclusive sources of resistance or conflicts that have a persistent influence on issues such as an adolescent’s metabolic control [26]. Although parents mentioned that they want to give their children adolescent independence, they also reported having fear and anxiety regarding the possibility that they might not manage their diabetes properly [27]. The occurrence of family crises was another factor that impaired parents’ coping with their children’s diabetes. Stanhope and Lancaster (2004) believed that family crises occur when family members cannot cope with an event and their organization and function is impaired.

The findings of this study suggested that toleration and management of difficult experiences may have both positive and negative impacts on the parents’ coping. Beasley and colleagues (2003) indicated that some people experience a high level of life tension without experiencing compromised physical or psychological well-being. Cognitive hardiness is a significant variable in the life tension/psychological and physical health equation. Having characteristics indicative of strong individual character, such as high hopes, may serve as a protective factor against psychological distress among mothers of very young children with diabetes [28]. It seems that stress management training courses for parents can increase their control or influence them to reduce the negative effects of the stress induced by diabetes in children.

Economic status was another factor that affected parents’ coping with their children’s diabetes. Monaghan and colleagues (2009) found that families with low incomes experienced more parental anxiety about their children’s metabolic control. Mothers of low-income families found it difficult to cope with the stress associated with their children’s diabetes, becoming more upset and experiencing higher levels of symptoms of anxiety and depression [29]. Since parents with lower socioeconomic status have more difficulties in coping with their children’s illnesses, it is necessary to provide them with support, such as governmental and public assistance.

In this study, the cost of treatment was another factor that influenced parents’ coping with their children’s diabetes. Pihoker and colleagues (2009) expressed that the costs of diabetes care have increased significantly in the past 10 years. The entire cost of diabetes per affected person in poor countries is more than twice the gross per capita national income [30]. Participants believed that the support provided by their support system is inadequate. Pihoker and colleagues (2009) believed that in some countries, health care or health insurance systems to decrease the raised price of diabetes care are bearing in mind or that they have already limited the use of newer technologies to stand up to 100% of the cost. Because diabetes imposes a considerable expense on families and causes concern among parents, the enhancement of insurance systems by governmental agencies for the further support of this group is essential.

Parental participation in care also affected parents’ coping with their children’s diabetes. Guthrie and colleagues (2003) commented that even though mothers often perform most care for children with diabetes, fathers are required to be involved, both to uphold mothers and to display to the children that their lack of participation in daily management does not signify refusal or a lack of caring. Despite the fact that fathers’ involvement in the care of their children with diabetes is low, their support of mothers and children with diabetes is necessary [7]. Although most of the time, fathers are not the initial caregivers or managers of usual daily care, their involvement with their families may be connected with important disease-management consequences.

The results of this study indicated that the coping strategies used by parents were different. Regardless of the strategies used by parents, they were concerned about their children’s futures. A study from Iran revealed that parents’ worries about their children’s diabetes were categorized into three aspects: concerns about their children’s future, the nature of diabetes and diabetes care, and social problems [14]. The understanding of parental concerns by nurses can improve parents’ communication with professional caregivers and their participation in the management of their children’s diabetes. Despite significant changes that have taken place in the last three decades in Iran (entailing increased employment and education among women), traditional gender-related roles are still generally preserved, and mothers are usually more responsible than fathers for taking care of the children. These findings necessitate planning for more fathers to participate in the management and monitoring of their children’s diabetes.

## Conclusion

We conclude that mediating factors in the parental coping process with their children’s diabetes consisted of four main categories: patient’s cooperation, crises and experiences, economic challenges, and parental participation in care. It seems that reinforcing positive features, such as support, warmth, and good relationships among family members, are highly important in facilitating cooperation between the patient and his/her family. Parents’ coping process with their child’s diabetes can be eased by psychological support such as psychological counseling, especially during situational crises; involvement of charities and the strengthening of insurance systems by the governmental sector in order to reduce the costs associated with juvenile diabetes; strengthening family confidence, positive attitudes, ability to manage stress, and accountability; and participation of both parents in managing the situation.

### Limitations of the study

This study was conducted using parents who were referred only to two diabetes centers. This limitation could influence the transferability of the study.

## Competing interests

The authors declare that they have no competing interests.

## Authors’ contributions

Study Design: FO, NM, HE. Data Collection: HE. Data Analysis: FO, NM, HE. Manuscript Writing: FO, NM, HE. Both authors read and approved the final manuscript.
